# Grandparenting practices and their association with physical and mental well-being among older adults with chronic diseases in Malaysia: A cross-sectional study

**DOI:** 10.51866/oa.586

**Published:** 2024-11-12

**Authors:** Sajaratulnisah Othman, Vinvie Wei Huo Hee, Clarice Jing Wen Ng, Julia Suhaimi

**Affiliations:** 1 MBBS, MFamMed, PhD, Department of Primary Care, Medicine, Faculty of Medicine University of Malaya, Kuala Lumpur, Malaysia. Email: sajar@um.edu.my, sajaratul@ummc.edu.my; 2 MD, MFamMed, Klinik Kesihatan Jalan Macalister, Penang, Malaysia.; 3 MBChB, University of Glasgow, United Kingdom, UK.; 4 MBBS, MFamMed, Department of Primary Care Medicine, Faculty of Medicine, Universiti Malaya, Malaysia.

**Keywords:** Grandparents, Elderly, Chronic diseases, Health

## Abstract

**Introduction:**

There is limited understanding of the association of grandparenting with the wellbeing of older adults with chronic diseases. This study aimed to examine grandparenting practices and their association with physical and mental well-being among older adults.

**Methods:**

The cross-sectional study was conducted at an outpatient clinic in a tertiary hospital in the Klang Valley, Malaysia. A total of 421 older patients with grandparenting experience were interviewed. The SF-12 Version 2.0 Health Survey was utilised to assess mental and physical wellbeing. Sociodemographic information, chronic disease care and grandparenting practices were evaluated to investigate any potential relationship with physical and mental well-being.

**Results:**

Of the participants, 80% were aged 60-74 years; 62.5% were women; and 81.4% completed secondary education. More than half of the participants voluntarily took on the role of grandparenting. The participants showed an impaired physical function but a preserved mental wellbeing. Never missing regular medication due to grandparenting and taking up grandparenting based on the circumstances were related to mental health. However, no factor was significantly associated with physical well-being.

**Conclusion:**

Two factors are linked to improved mental well-being. In contrast, there is no significant relationship found between a decline in physical health and grandparenting practices. Further research is needed to determine the causal relationship between physical health challenges and grandparenting practices.

## Introduction

The increasing life expectancy in Malaysia has led to a significant shift in population structure, resulting in a larger proportion of older adults. According to the Department of Statistics Malaysia, the percentage of the population aged 65 years and above in 2021 was 7.0%, qualifying Malaysia as an ageing nation by the United Nations’ definition. This trend is accelerating, with the ageing population projected to reach over 14.5% in 2040.^1,2^ This rapid demographic shift, combined with improved healthcare access and healthier lifestyles, necessitates a deeper understanding of the factors influencing the wellbeing of older adults practising grandparenting, a role that is becoming increasingly common and important in Malaysian society.

Grandparenting, defined as “the act of caring for (or involvement with) one’s grandchildren or the children of one’s children”,^3^ has become a crucial aspect of family dynamics in the modern world.

The rise of dual-earner households and high rates of family breakdown have led to many adults relying on their parents to care for their children. In this context, grandparents have become indispensable in raising the younger generation, playing a vital role in the family structure.

Ageing is a natural part of life. As people age, they gain skills, experience and wisdom, but ageing is also often associated with a decline in health. Studies by the Survey on Health, Ageing and Retirement in Europe have shown that the risk of chronic diseases increases with age. In 2017, 37% of individuals aged 65 years and above were reported to have at least two chronic diseases across European Union countries. Among individuals aged 80 years and above, the percentage of women and men reported to have multiple chronic diseases across EU countries was 56% and 47%, respectively.^4^ In Malaysia, local research has shown that the percentage of older adults (aged 50 years and above) having two or more chronic diseases is 40.6%, with hypertension and dyslipidaemia being the two most common chronic diseases.^5^

The increase in grandparenting practices can have various health impacts, making it a significant healthcare concern. Previous research has reported mixed findings regarding the impact of grandparenting on the health of older adults. Some studies have shown that the decline in the health status of grandparents is proportional to the time spent on grandparenting. Research conducted in the United States of America (USA) has indicated that custodial grandparents who provide full-time care for their grandchildren are more likely to have limitations in their daily activities and lower levels of satisfaction with their health.^6^ Musil and Ahmad showed that primary caregiver grandparents reported poorer self-assessed health than their partial or non-caregiver counterparts.^7^

On the contrary, a European study found that grandparents experienced health benefits regardless of the level of intensity in looking after their grandchildren.^8^ A study among grandmothers in Turkiye reported that grandparenting positively affected the quality of life, perception of health and level of depression, except among custodial grandmothers.^9^ A study performed in China showed that the effect on older adults’ health was not only affected by the intensity and duration of grandchild care but also shaped by individual characteristics, cultural norms and living arrangements (co-residing or otherwise).^10^

Older adults generally require excellent health and social care, thus increasing the country’s public expenditure. With the increase in the ageing population and the number of grandparents practising grandparenting in Malaysia being inevitable, promoting healthy ageing is crucial for maintaining the quality of life among older people. Investigating the association of grandparenting with older adults’ well-being and understanding the factors that can influence the well-being of older adults who are grandparenting are essential for healthy ageing. However, more extensive local data on the relationship between grandparenting and older adults’ health are needed.^11^ This study aimed to explore grandparenting practices and their association with physical and mental wellbeing among older adults.

## Methods

This cross-sectional study was conducted at an outpatient clinic in a tertiary hospital in the Klang Valley, Malaysia. Older adults aged 60 years and above who were diagnosed with chronic diseases, were caring for their grandchildren and could understand Malay or English were included. Conversely, older adults who were physically incapacitated or had a mental or cognitive disorder were excluded. Mental health was screened by asking whether older adults had any mental health conditions or were under any psychiatric follow-up.

Data were collected from 17 September to 15 November 2021 via convenience sampling. This sampling approach was selected due to challenges in recruiting participants during the pandemic, with many older adults with stable medical conditions having their appointment dates rescheduled as a measure to reduce the risk of COVID-19 transmission by staying at home. Few older adults were tech-savvy enough to accept the teleconsultation offered in the clinic. Furthermore, face-to-face data collection was considered a better screening option for cognitive disorders. Collecting large samples in a limited amount of time was also needed; hence, convenience sampling was used. However, this sampling method has limitations, one of which is the risk of bias. Our recruitment process was designed to minimise potential sources of bias. The sample size was determined based on a margin of error of ±5. Informed consent was obtained from all participants, which involved an explanation that there were no correct or incorrect answers. We also ensured that participants were fully informed about the confidentiality measures in place and that there would be no repercussions for choosing to participate or decline, thereby upholding the highest ethical standards in our research.

We approached potential participants while they were waiting for their doctor’s consultation. Those who fulfilled the study criteria and provided informed consent proceeded to answer a hardcopy questionnaire independently. For illiterate participants, trained interviewers read the questionnaire aloud, offering assistance without adding their opinions or explanations to minimise bias.

The sample size was computed as previously described in a study conducted in 2007.^12^ The mean and standard deviation (SD) were derived from Table 4 of that study. Based on these values and using the equation N=(1.96×SD/ME)^2^, we calculated the minimum required sample size to estimate the mean in each domain within a margin of error of ±5. The largest minimum sample size derived was 418. This size accounted for a 20% (n=70) adjustment for potential non-respondents, ensuring the validity and reliability of the final sample size. Out of 450 participants approached, 421 were successfully recruited, yielding a response rate of 93.6%. The data of all 421 participants were used for analysis. The interviewer doublechecked each questionnaire for completeness upon submission, ensuring that all data were available for analysis.

The questionnaire used in this study has both Bahasa Malaysia and English versions and consists of the following five sections:

1. Sociodemographic characteristics: This section collects data on age, sex, ethnicity, educational level, marital status and monthly household income.
2. Grandparenting practices: This section gathers details about the number and age of each grandchild under care, the duration of care per week, whether the grandchild stays overnight without the presence of parents, the source of monthly income, the total number of household members and the reason for grandparenting (voluntary, situational or mixed).
3. Chronic disease care: This section assesses the type of medical condition, any missed clinic appointments due to grandparenting and any missed medication due to grandparenting.
4. Perception of grandparents on grandparenting: This section includes three subjective questions regarding enjoyment, stress and health decline while grandparenting, using a 5-point Likert scale that scores items from 1 to 5. The total perception score is obtained by summing the Likert scale scores from the three questions, with 3 points being the lowest possible score and 15 points being the highest possible score. As the median is 10 points, scores of 10 points and above are considered to indicate positive perceptions, and scores of 9 points and below are deemed to indicate negative perceptions.
5. SF-12 Version 2.0 Health Survey (SF12v2): This 12-item questionnaire assesses overall physical and mental functions across eight health domains: physical function, role limitation – physical, bodily pain, general health, vitality, social function, role limitation – emotional and mental health.

The questionnaire underwent face and content validation by an expert panel comprising two family medicine specialists and two geriatricians to improve its accuracy and adequacy. The questionnaire was also pilot-tested among 25 older adults caring for grandchildren in the same setting as the actual study to examine the feasibility of the study, the recruitment process and the comprehensibility and appropriateness of the questionnaire. Based on participants’ feedback, minor changes were made to the format and font size of the questionnaire. Participants required approximately 15 minutes to complete the questionnaire, and all reported that it was easily understood.

Internal consistency was assessed using Cronbach’s alpha coefficients for the perception of grandparents on grandparenting section. The calculated Cronbach’s alpha coefficient was 0.47, which was considered unsatisfactory. Ideally, a Cronbach’s alpha coefficient of 0.60 or higher is considered acceptable. However, slightly increasing the number of items would lead to an acceptable Cronbach’s alpha coefficient.^13^ Since the perception of grandparents on grandparenting section has only three items and was based only on a sample size of 25, the Cronbach’s alpha coefficient was low. This can be justified by the increase in the Cronbach’s alpha coefficient to 0.59 when calculated using the full sample size of 421. Therefore, despite the low value, the data collected were still analysed.

Tests and re-tests were conducted to assess the internal consistency of the questionnaire. Kappa statistics and intraclass correlation coefficients (ICCs) were calculated. The kappa statistics exceeded 0.40, indicating a strength of agreement ranging from almost perfect to perfect. The ICCs exceeded 0.50, showing a strength of agreement ranging from good to excellent.

The definition of the types of grandparenting used in this study was based on the study by Fuller-Thomson and Minkler, categorising grandparenting into four types: extensive, intermediate, occasional and primary caregivers.^14^ The types of chronic diseases included in this study were diabetes mellitus, hypertension, dyslipidaemia, osteoporosis and others. The first four types of chronic diseases were highlighted based on NHMS 2018 data, which showed a high prevalence of diabetes mellitus, hypertension and dyslipidaemia among Malaysian older adults.^15^ In addition, osteoporosis is also a significant public health problem in developing countries as the population of older adults is increasing. A study conducted in Kuala Lumpur, Malaysia, also showed that many male and female adults aged 50 years or above had suboptimal bone health. We excluded cancer, stroke and Parkinson’s disease from our analysis, as these conditions are likely to adversely affect cognitive behaviour, potentially influencing the focus of our research findings.

Household income was categorised into three groups: the lowest 40% (B40), the middle 40% (M40) and the highest 20% (T20). The B40, M40 and T20 groups included households earning less than RM 4850, RM 4850-10,959 and RM 10,960 and above, respectively.^16^

Physical and mental health were assessed based on how older adults felt about their health using the SF12v2. The SF12v2 is a valid and shorter alternative version of the SF-36, comprising only 12 questions that measure eight health domains including the overall physical and mental functions. 17 It has been proven helpful in conducting surveys for general and specific populations.^18-26^ The SF-12 is preferable to other tools, as it is short yet still valid and has fewer questions, making it easier to answer, especially among older adults. In addition, this questionnaire was adapted from previous studies on grandparents raising grandchildren.^9,12^ The SF12v2 was developed after 10 years of research, featuring a modified scale. The number of questions remained unchanged, but the questions were simplified and abbreviated. The eight health domains include the following: physical function, role limitation - physical, bodily pain, general health, vitality, social function, role limitation – emotional and mental health. Each domain uses different rating scales. Physical function has two items rated on a 3-point Likert-type scale: 1=yes, limited a lot; 2=yes, limited a little; 3=no, not limited at all. Bodily pain has one item scored on a 5-point Likert-type scale: 1=not at all, 2=a little bit, 3=moderately, 4=quite a bit, 5=extremely. General health has one item scored on a 5-point Likert-type scale: 1=excellent, 2=very good, 3=good, 4=fair, 5=poor. Vitality and social function each have one item, whereas mental health and emotional and physical role limitations each have two items; all are measured using a 5-point Likert-type scale: 1=all of the time, 2=most of the time, 3=some of the time, 4=a little of the time, 5=none of the time.

Each score is calculated using the SF12v2 manual guide as a reference to determine the fundamental values for the mental and physical components. The scores for the mental component summary (MCS) and physical component summary (PCS) are calculated by adding specific constant numbers to these two components using established data from the 1990 US general population. This ensures the validity and reliability of the process. The first step involves computing aggregate scores for the physical and mental components using the factor score coefficients and z-scores from each of the eight health domains. This is followed by multiplying each SF12v2 health domain scale z-score by its respective physical factor score coefficient; the resulting eight products are then summed. Similarly, an aggregate mental component score is obtained by multiplying each health domain scale z-score by its respective mental factor score coefficient and then summing the resulting eight products. The z-score is computed by subtracting each mean health domain scale score in the US general population from the 0–100 score for that scale and then dividing the difference by the SD for that scale. The T-score method is used to interpret the PCS and MCS measures of the SF12v2. This method involves a transformation process using means and SDs derived from general population norms and factor score coefficients based on the 1990 US general population norms.^17^ The transformation process ensures that the PCS and MCS have a mean of 50 and an SD of 10 in the 2009 US general population. The T-score is then calculated by multiplying each aggregate component scale score by 10 and adding 50 to the resulting product.

In this study, a group mean scale score was used. The internal consistency for the PCS and MCS measures was found to be 0.92 and 0.88, respectively, indicating good reliability. This calculation was performed using the covariance matrix of the SF12v2 health domain scales and the physical and mental factor score coefficients from the 2009 US general population.^27,28^ A group mean scale score below 47 indicates a below-average health status, while a score of 47 and above indicates an above-average health status.^27^

The data were cleaned and analysed using the ‘IBM SPSS Statistics for Windows, version 26 (IBM Corp., Armonk, N.Y., USA).^28^ The categorical data were analysed using descriptive statistics and presented as frequencies and percentages. The continuous data were described as means or medians depending on whether they were normally distributed or skewed. Independent factors with a significance level of ≤0.25 in the univariate analysis were included in the multivariate analysis. A multiple linear regression analysis was conducted to examine the association between the independent variables (e.g. age, marital status and diabetes mellitus) and the dependent variables (e.g. physical and mental well-being). The assumptions of multiple linear regression, including linearity, independence of errors and normality of residuals, were assessed and met. A P-value of <0.05 was considered statistically significant.

## Results

Table 1 summarises the participants’ sociodemographic characteristics and medical conditions. Most participants (80.5%) were aged 60-74 years, with a mean age of 69.6±5.8 years. Around two-thirds (62.5%) were women.

Approximately 35.6%, 39.4% and 22.6% were Malay, Chinese and Indian, respectively; conversely, 2.4% had other ethnicities. Only 3.3% of the participants had no formal education, and 86.8% were married. Nearly half of the participants (49.2%) fell into the B40 household income group. The most common chronic medical conditions among the participants were hypertension (75.1%) and dyslipidaemia (67.9%). About 98% and 95.2% of the participants reported that grandparenting did not result in missed clinic follow-up and medication, respectively.

**Table 1:** Sociodemographic characteristics and medical conditions of the older adults caring for their grandchildren (N=421).

Variable	Category	n	%	Mean (SD)
Age				69.6 (5.8)
	60-74 (Min: 60) years	339	80.5	
	>75 (Max: 89) years	82	9.5	
Sex				
	Male	158	37.5	
	Female	263	62.5	
Ethnicity				
	Malay	150	35.6	
	Chinese	166	39.4	
	Indian	95	22.6	
	others	10	2.4	
Educational level				
	No formal education	14	3.3	
	Primary education	63	15.0	
	Secondary education	190	45.1	
	Tertiary education	153	36.3	
Marital status				
	Married	364	86.5	
	Divorced/separated	5	1.2	
	Widowed	52	12.4	
Household income				
	T20	30	7.1	
	M40	162	38.5	
	B40	207	49.2	
Chronic medical conditions^*^				
	Diabetes mellitus	183	43.5	
	Hypertension	316	75.1	
	Dyslipidaemia	286	67.9	
	Osteoporosis	44	10.5	
	Others (ischaemic heart disease, gout, glaucoma or osteoarthritis)	77	18.3	
Missed clinic follow-up due to grandparenting				
	No	413	98.1	
	Yes	8	1.9	
Frequency of missed medication due to grandparenting				
	Never	401	95.2	
	Less than once per week	13	3.1	
	Once a week	4	1.0	
	Every day	3	0.7	

SD: Standard deviation

* Multiple responses permitted

Table 2 summarises the different types of grandparenting practices and the reasons for grandparenting. The majority of the participants were occasional caregivers (34.4%), followed by intermediate caregivers (28.7%), primary caregivers (20.7%) and extensive caregivers (16.2%). About 54.9% of the participants reported volunteering as their reason for grandparenting; 20.4% cited situational reasons; 20.2% mentioned a combination of volunteering and situational reasons; and 5.7% reported other reasons such as cultural factors, responsibility and living arrangements. The participants generally perceived grandparenting positively. Nearly half of the participants reported immensely enjoying grandparenting (49.17%) and feeling no stress at all (49.9%). Additionally, 79% stated that grandparenting did not have a negative impact on their health. The total perception score for grandparenting indicated a positive outlook; with a mean score of 12.947 (SD=2.0534) out of a possible 15 points (median=10). The minimum score of 5 and the maximum score of 15.

**Table 2:** Grandparenting practices (N=421).

Practice	n	%
Types of grandparenting		
Occasional caregivers^a^	145	34.4
Intermediate caregivers^b^	121	28.7
Extensive caregivers^c^	68	16.2
Primary caregivers^d^	87	20.7
Reason for grandparenting^#^		
Voluntary	231	54.9
Mixed (voluntary and situational)	85	20.2
Situational	86	20.4
Others^*^	24	5.7

a Duration of care: 1–9 hours per week and/or overnight for <6 nights per year without the presence of parents

b Duration of care: 10–29 hours per week and/or 7–89 nights per year without the presence of parents

c Duration of care: At least 30 hours per week and/or at least 90 nights per year without the presence of parents

d Custodial caregiver

* Culture, responsibility or living together

# More than one reason selected per participant

Table 3 shows the physical and mental component scores in the SF12v2. The mean physical and mental component scores were 42.8739 (SD=7.91830) and 59.3264 (SD=7.96542), respectively, indicating that the participants generally had an impaired physical well-being but a preserved mental function.

**Table 3:** Physical and mental well-being scores in the SF12v2 (N=421).

Well-being	Minimum	Maximum	Mean	SD
PCS	15.15	61.18	42.8739	7.91830
MCS	33.70	77.42	59.3264	7.96542

PCS: Physical component summary (score below 47 indicates impaired function)

MCS: Mental component summary (score below 47 indicates impaired function)

Table 4 shows the results of the multiple linear regression analysis on the association of the sociodemographic characteristics and grandparenting practices with the physical well-being of the participants. The analysis showed no significant predictor for physical well-being. Moreover, there was no collinearity, as all variance inflation factor (VIF) values were <5.

**Table 4:** Coefficients for physical well-being.

Model	Unstandardised coefficients		Standardised coefficients	t	Sig.	Collinearity statistics	
	B	Std. error	Beta			Tolerance	VIF
1 (Constant)	52.082	6.682		7.795	0.000		
Age	-0.106	0.076	-0.078	-1.393	0.165	0.767	1.303
Sex	-1.128	0.872	-0.069	-1.293	0.197	0.854	1.171
Educational level	0.907	0.565	0.090	1.605	0.109	0.770	1.299
Household income	-0.519	0.690	-0.041	-0.753	0.452	0.808	1.237
Diabetes mellitus	-1.095	0.807	-0.068	-1.356	0.176	0.952	1.050
Hypertension	-1.096	0.926	-0.060	-1.184	0.237	0.955	1.047
Osteoporosis	-2.264	1.349	-0.086	-1.679	0.094	0.910	1.098
Voluntary grandparenting	1.162	0.806	0.072	1.441	0.151	0.953	1.049
Number of grandchildren aged 5 years and below under care	-0.632	0.955	-0.039	-0.661	0.509	0.690	1.449
Number of grandchildren aged 13 years and above under care	-1.465	1.010	-0.084	-1.451	0.148	0.720	1.389
Other sources of income	0.528	0.989	0.028	0.533	0.594	0.854	1.171
Living with children and grandchildren	0.791	0.856	0.048	0.924	0.356	0.892	1.121

Table 5 summarises the results of the multiple linear regression analysis on the association of the sociodemographic characteristics and grandparenting practices with the mental well-being of the participants. There was no collinearity, as all VIF values were <5. The analysis showed that the frequency of missed medication due to grandparenting and a situational reason for grandparenting were significantly associated with mental well-being.

**Table 5:** Coefficients for mental well-being.

Model	Unstandardised coefficients		Standardised coefficients	t	Sig.	Collinearity statistics	
	B	Std. error	Beta			Tolerance	VIF
1 (Constant)	61.640	7.507		8.211	0.000		
Age	0.092	0.072	0.067	1.282	0.201	0.804	1.244
Marital status	-2.125	1.155	-0.091	-1.840	0.066	0.896	1.116
Diabetes mellitus	-1.393	0.786	-0.087	-1.773	0.077	0.922	1.085
Other medical conditions	-1.765	0.988	-0.086	-1.786	0.075	0.958	1.044
Missed clinic follow-up due to grandparenting	-1.919	2.792	-0.033	-0.687	0.492	0.962	1.039
Frequency of missed medication due to grandparenting	-2.972	0.903	-0.157	-3.291	0.001	0.971	1.030
Voluntary grandparenting	1.144	0.929	0.072	1.232	0.219	0.655	1.527
Grandparenting based on the situation	-2.252	1.133	-0.114	-1.987	0.048	0.670	1.493
Two grandchildren under care	0.683	0.936	0.041	0.730	0.466	0.686	1.458
Three or more grandchildren under care	-0.385	0.954	-0.023	-0.404	0.686	0.681	1.469
Number of grandchildren aged 5 years and below under care	-0.459	0.837	-0.028	-0.549	0.583	0.824	1.214
Other sources of income	0.882	0.884	0.048	0.998	0.319	0.969	1.032

## Discussion

The participants were mostly Chinese (39.4%) and women (62.5%). A possible explanation is the longer life expectancy of Chinese and female populations based on Malaysia’s health and population statistics. This provides Chinese women with greater opportunities to become grandparents.^29^

This study found that the participants were physically impaired. However, poor physical health could result from reasons other than grandparenting. The participants were patients waiting to seek treatment at the clinic, suggesting that their physical well-being could have been affected by their unoptimised health issues during the survey. Conversely, studies from other countries that reported positive physical well-being likely drew data from national health survey databases rather than from clinic settings.^8, 9^

The participants in the present study showed a preserved mental well-being, corresponding to most reports from other countries such as Korea and China.^30,31^ In Malaysia, where caring for younger children is still viewed as a shared family responsibility,^32^ grandparenting does not cause much mental stress to Malaysian grandparents compared to involuntary grandparenting, as observed in the USA, where people do not take on grandparenting responsibilities unless required.^33^

Due to the COVID-19 pandemic lockdown, which forced parents to work from home, many parents chose to care for their children by themselves to reduce the risk of COVID-19 transmission to their grandparents. This corresponds to the results of this study, wherein most participants were occasional caregivers. In contrast, most participants in the study by Fuller-Thomson and Minkler were intermediate caregivers.^14^

The majority of the study participants (54.9%) volunteered for grandparenting, while 20.4% reported grandparenting based on the situation. Similarly, Alavi et al. showed that some older adults practised grandparenting as a hobby and due to their interest, indicating a more volunteer-based practice. Some participants reported grandparenting based on their situation, such as family finances and young parents pursuing education.^34^

Smorti et al. reported that grandparents exhibited positive emotions towards their relationships with their grandkids.^35^ The current study similarly showed that the total perception score was 12.947 (SD=2.0534) out of 15 points, indicating a positive perception. This could be because most participants were occasional or intermediate caregivers and therefore had some spare time to enjoy themselves. Furthermore, this study found no significant factors associated with physical health.

The present study found that the situational reason for grandparenting and the frequency of missed medications due to grandparenting were the two significant factors associated with mental well-being. Wang et al. reported that caregiving burden could lead to stress-related depression and, ultimately, missed medication.^36^ In contrast, this study showed that nearly all participants (95.2%) never missed medication due to grandparenting. This could be because more than 50% of the participants were occasional or intermittent caregivers, which likely reduced caregiver burden, preserved their mental function and prompted them to never miss their medication. Furthermore, most participants in this study had a source of income, a high educational level and access to medical care.

In the country, older adults are exempt from outpatient services and health charges. The participants who cited a situational reason for grandparenting primarily referred to grandparenting as an obligation. According to McGarrigle et al., the reason for grandparenting impacts the well-being of grandparents, and those who practise grandparenting out of obligation report lower happiness levels.^37^ This finding corresponds to the results found in this study. The majority of the participants had a preserved mental well-being, which could be because they did not practise grandparenting based on their situation.

To the best of our knowledge, this study is the first to examine grandparenting practices in relation to grandparents’ health in Malaysia. It also analysed the factors associated with grandparents’ health. This study is significant because it was sufficiently powered by an adequate sample size. The findings can be used as a reference for future studies in this field. However, there are several limitations in the study design, which may impact the conclusions drawn. With the study conducted using convenience sampling, the results may be generalised only to some caregiver grandparents in Malaysia. Hence, the findings primarily represent the participants in this study, as evidenced by the higher recruitment rate of female and occasional caregivers compared to male and other caregivers. Furthermore, grandparents who missed their clinic appointments due to grandparenting were excluded from the study. In addition, the questionnaire used in this study underwent only face and content validation and stability and reliability tests.

Given the unavailability of some data due to limited access, the findings relied on the answers provided by the participants. Moreover, ensuring the security, privacy and protection of healthcare data is critical for all healthcare personnel and institutions^37,38^; hence, the accuracy of the medical conditions could not be verified if the participants did not grant permission due to confidentiality concerns. This study adopted a cross-sectional design; thus, the results can only be considered exploratory. Further studies are required to compare results and determine the factors contributing to impaired physical well-being in grandparenting older adults, given that this study found no significant factor affecting the physical wellbeing of the participants.

## Conclusion

In conclusion, although this study found that the participants’ physical well-being was impaired, the analysis did not reveal a significant factor associated with physical impairment among the participants. This suggests that there may be other factors contributing to their physical health issues, warranting further investigation. Conversely, the mental well-being of the participants was preserved. Never missing medication due to grandparenting and taking up grandparenting based on the situation were significantly associated with mental well-being. These findings highlight the importance of considering the mental health implications of grandparenting and the need for policies that support older adults in their caregiving roles.

## Data sharing statement

All data generated and analysed in the study are available upon reasonable request.

**How does this paper make a difference in general practice?**

- The study emphasises the importance of considering the mental well-being of older adults with chronic diseases who practise grandparenting.
- The analysis identifies two factors linked to improved mental well-being: never missing regular medication due to grandparenting and taking up grandparenting based on the situation.
- The findings can inform general practice approaches to support older adults with chronic diseases who are involved in grandparenting.
